# Risk prediction models for depression in cancer survivors: A systematic review and meta-analysis

**DOI:** 10.1097/MD.0000000000043978

**Published:** 2025-08-22

**Authors:** Haonan Xu, Wenqiang Tang, Yuwen Liang, Kunyuan Gan, Xinlan Liu, Xiongxin Yang, Xiaobo Du

**Affiliations:** a Department of Oncology, NHC Key Laboratory of Nuclear Technology Medical Transformation (Mianyang Central Hospital), Mianyang Central Hospital, School of Medicine, University of Electronic Science and Technology, Mianyang, People’s Republic of China; b Department of Oncology, Affiliated Hospital of North Sichuan Medical College, Nan Chong, People’s Republic of China; c Sichuan Clinical Research Center for Radiation and Therapy, Mianyang, People’s Republic of China; d Department of Hepatobiliary Surgery, Affiliated Hospital of North Sichuan Medical College, Nan Chong, People’s Republic of China.

**Keywords:** cancer survivors, depression, prediction model, risk, systematic review/meta-analysis

## Abstract

**Background:**

To systematically evaluate the risk prediction models for depression in cancer survivors, so as to provide guidance for establishing and improving models.

**Methods:**

CNKI, Wanfang Database, Sinmed, PubMed, Web of Science, The Cochrane Library, and Embase were searched for studies on cancer survivors published before July 1, 2024. The prediction model risk of bias assessment tool was used to evaluate the quality of the studies on the prediction model, and the Stata 15 software was used to conduct a meta-analysis of the predictive variables for the establishment of the model.

**Results:**

Seven articles were included, and 7 prediction models from 7 studies were included in this review. The results of the PROBAST evaluation showed that all the 7 studies were at high risk of bias, but the applicability was good. The area under the curve (AUC) = 0.685 to 0.885. All the included studies were validated internally, using the Bootstrap test, Hosmer–Lemeshow test, and temporal validation, respectively. The random effects model was used to perform the meta-analysis on AUC and C-index. The meta-analysis result of AUC was 0.705 (95% CI: 0.691–0.718), I^2^ = 92.6% (*P* < .001), *P* < .0001. The meta-analysis of C-index was 0.833 (95% CI: 0.814–0.852), I^2^ = 66.5% (*P* = .030), *P* < .0001.

**Conclusions:**

At present, risk prediction models for depression in cancer survivors are still in the research and development stage, and the overall quality of research needs to be further improved.

## 1. Introduction

With the development of modern medicine, the biomedical model has been replaced by the biopsychosocial medical model. Malignant tumor not only brings patients obvious discomfort, but also imposes a huge mental pressure on them. Cancer patients experience anxiety and fear of death for a long time, along with mental pressure, and treatments such as surgery, radiation, and chemotherapy for tumors can also cause physical discomfort and changes in appearance. These factors combined are likely to cause psychiatric disorders, with cancer-related depression being the most common.^[1]^ Depression, also known clinically as depressive disorder, is characterized by significant and persistent low mood. In the early stage of depression, patients experience low mood, which gradually progresses to depression and low self-esteem, resulting in a certain degree of world-weariness. This condition easily induces suicidal behavior or ideation, imposing great psychological pressure on the physical and mental health of patients. Cancer patients face not only the physiological threat of disease, but also social, emotional, psychological, and physical pressures, which can cause serious emotional problems.^[2–4]^ The American Cancer Society’s psychological research indicates that 13% of cancer patients experience severe depression, while 34% have varying degrees of depression.^[5]^ Studies show that depression is one of the most common psychological disorders in cancer patients, and that depression not only reduces their quality of life, but also significantly affects disease recovery.^[6]^ Therefore, analyzing risk factors for depression in cancer patients and implementing proper management are of great significance for their care. Prediction models use mathematical models combined with predictors to calculate the probability of an event’s outcome.^[7]^ In recent years, many researchers domestically and internationally have established depression risk prediction models for cancer survivors.^[8–14]^ However, there is a lack of comprehensive comparative studies on the model construction process, performance, and data sample bias, and it is not clear whether the tool can be applied in clinical practice. Therefore, this study aims to systematically evaluate depression risk prediction models for cancer survivors from domestic and international research, providing guidance for clinical medical personnel in screening for depression risk in cancer survivors.

## 2. Methods

### 2.1. Search strategy

We conducted a comprehensive search targeting both Chinese and English databases, retrieve database including China National Knowledge Infrastructure (CNKI), Wanfang Database, Sinmed, PubMed, Web of Science, The Cochrane Library, and Embase. The retrieval time was from the beginning of database construction to July 1, 2024. The search keywords were as follows: “cancer,” “depression,” “risk prediction model,” “risk factor,” “predictor,” “model,” and “risk score.” Because this is a meta-analysis, ethical approval was not necessary.

### 2.2. Inclusion and exclusion criteria

The inclusion criteria for studies were: the object of study for cancer survivors; the research content for the building and/or verify the forecast model of cancer survivors depression occur; the research design for retrospective study or prospective study; outcome indicators for cancer survivors depression happened; language is Chinese or English.

The exclusion criteria were: repeated publications; reviews, case studies, and conference abstracts; cannot get the original document.

### 2.3. Data extraction and quality evaluation

Two researchers (T.W.Q., L.Y.W.) assessed the eligibility of all retrieval studies and independently extracted relevant data. Extracted databases were then cross-checked between the 2 authors to rule out any discrepancy. Disagreements were resolved by consulting with a third investigator (X.H.N.). The following data were extracted independently for each study collected: author, year of publication, country, study type, patient type, main outcome, number of patients, and model parameters. Our investigation process complied with the Preferred Reporting Items for Systematic Reviews and Meta-analyses (PRISMA) statement.

Prediction model risk of bias assessment tool (PROBAST) evaluation tools to assess risk of bias and applicability of the literature.^[15]^ The risk of bias of the model was measured from 4 perspectives: subjects, predictors, outcomes, and data analysis. The applicability evaluation was carried out from 3 perspectives, and the evaluation procedure was similar to the risk of bias analysis procedure. The evaluation criteria for different perspectives were 3 grades of “low,” “high,” and “unclear,” and the best model was selected according to the above characteristics. PROBAST can not only evaluate a single model, but also compare multiple models.^[15]^

### 2.4. Statistical analysis

Stata18 software (StataCorp LLC, College Station) was used to perform meta-analysis on the area under the curve (AUC) and C-index of the included models, and mean difference value and 95% CI were used as effect statistics. The Q test and I^2^ statistic were used to evaluate the heterogeneity of multiple studies. If *P* > .1 or I^2^ < 50%, inter-study heterogeneity was considered to be small, and fixed effect model was selected. If *P* ≤ .1 or I^2^ ≥ 50%, the inter-study heterogeneity was considered to be large, and further sensitivity analysis was performed. If the heterogeneity could not be eliminated, the random effect model was selected. *P* < .05 was considered statistically significant. Egger test was used to determine publication bias, and *P* > .05 indicated a low possibility of publication bias.^[16,17]^.

### 2.5. Literature screening and data extraction

Two researchers (L.Y.W. and Y.X.X.) screened the literature according to the inclusion and exclusion criteria. In case of disagreement, a third researcher (X.H.N.) was consulted. According to Moons et al^[18]^ check list for critical appraisal and data extraction for systematic reviews of predictive models prediction modeling studies (CHARMS), a data collection form, including: the first author, publication date, country, study type, follow-up time, location, outcome indicators, sample size of the study, method of constructing the model, number of models, AUC, and calibration method, verification method used in the model, influencing factors included in the prediction model, model presentation method, etc.

## 3. Result

### 3.1. Literature search results

**Figure 1.**
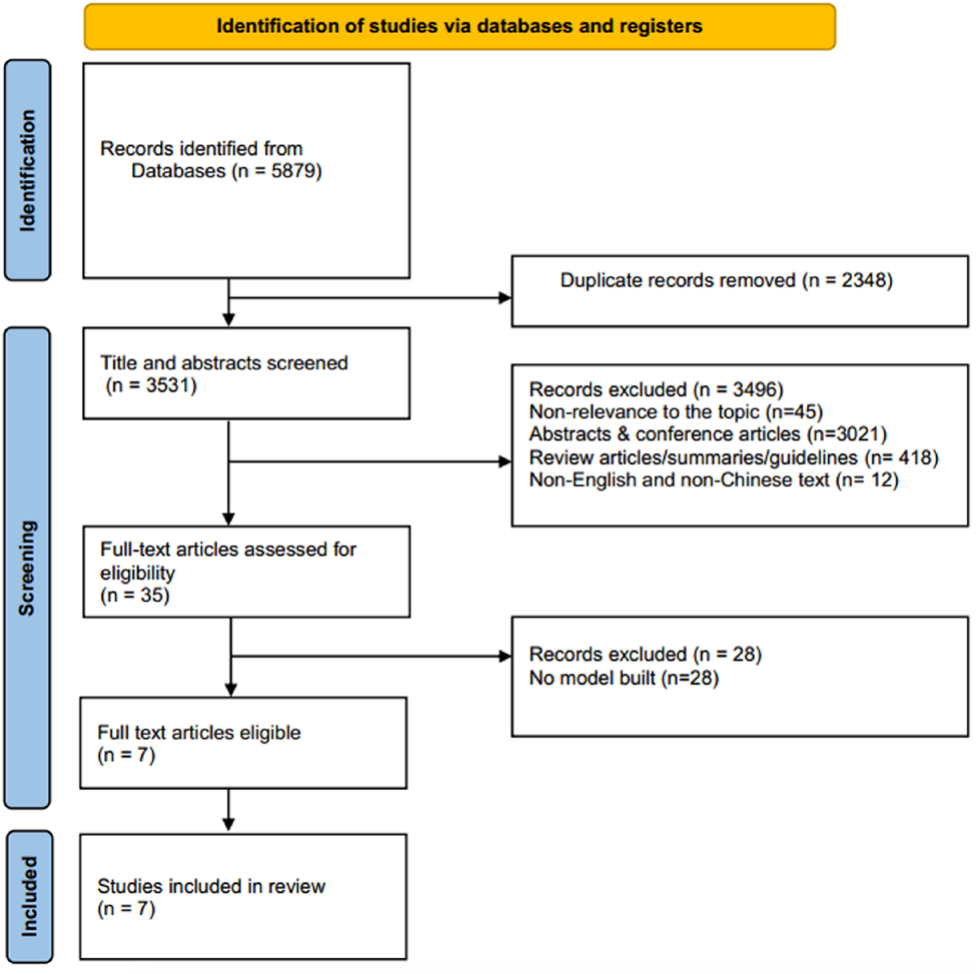
Illustration of the literature retrieval and screening process.

The results of the search and excluded literature are shown in Figure 1

### 3.2. The basic characteristics of risk prediction model

All the 7 articles were retrospective studies. The basic data of the included studies are shown in Table 1. Random forest and logistic regression were used in 1 study, and logistic regression was used in the remaining 6 studies. In the processing methods of continuous variables, 4 literatures transformed the continuous variables into binary variables to deal with them, and 3 of them maintained the continuity of the continuous variables. In terms of variable selection, 1 literature used stepwise regression analysis to screen variables, and the remaining literatures were based on univariate analysis and multivariate analysis. The AUC of the model features included in this study ranged from 0.685 to 0.885, and one of them reported a specificity of 66.67% and a sensitivity of 76.19%. Most of the included studies predict ability is higher (AUC > 0.7). In terms of model validation, all the included studies used internal validation, among which 5 studies used Bootstrap method, 1 used the Hosmer–Lemeshow test, and the other used temporal validation. The presentation form of the model was mainly nomogram analysis score, and the presentation form of the other paper was formula of risk score obtained by partial regression coefficient of each factor. Modeling situation of the included studies are shown in Table 2. Performance and presentation of the model are shown in Table 3.

**Table 1:** Overview of basic data of the included studies.

References	Country	Study design	Participants	Main outcome
Zou^[8]^	China	Retrospective Study	Accepted the surgical treatment of cervical cancer survivors, and no history of mental illness.	Hamilton Depression Scale (HAMD)
Jia^[9]^	China	Retrospective Study	Head and neck cancer survivors receiving antineoplastic therapy, and no history of mental illness.	Hospital Anxiety and Depression Scale (HADS)
Li^[10]^	China	Retrospective Study	Castrated prostate cancer survivors with no history of psychiatric disorders.	Self-rating Anxiety and Depression Scale (SAS/SDS)
Wang^[11]^	China	Retrospective Study	Breast cancer survivors.	A methylation-derived Depression Index (mDI)
Hu^[12]^	China	Retrospective Study	Colorectal cancer survivors.	The Hospital Anxiety and Depression Scale (HADS)
Zhu^[13]^	China	Retrospective Study	Thyroid cancer survivors undergoing thyroid surgery.	Distress Thermometer (DT)
Dong^[14]^	China	Retrospective Study	Gynecological cancer survivors and always did not suffer from anxiety, depression and other mental illnesses.	Minnesota Multiphasic Personality Tests (MMPI) scale

**Table 2:** Modeling situation of the included studies.

References	Modeling method	Selection of variables	Positive cases/sample size (%)	Continuous variable processing method	Final prediction
Zou^[8]^	Logistic regression mode	Single factor analysis, Multivariate analysis	58/200	Categorical variables	Poor economic conditions, Low educational level, If an unmarried, Hysterectomy, Cervical cancer middle-late.
Jia^[9]^	Logistic regression mode	Single factor analysis, Multivariate analysis	123/332	Continuous variable	Tumor stage, presence or absence of tracheal tube, functional status score, anxiety score, perceived social support score, negative coping score.
Li^[10]^	Logistic regression mode	Single factor analysis, Multivariate analysis	35/84	Categorical variables	Marital status, castration scheme, and postoperative Visual Analogue Scale (VAS) score.
Wang^[11]^	Logistic regression mode, random forest methods.	Stepwise Regression Analysis	35.7%	Continuous variable	Statistical difference of DNA Methylation between Promoter and Other body region (SIMPO).
Hu^[12]^	Logistic regression mode	Single factor analysis, Multivariate analysis	217/602	Categorical variables	Gender, personal status, income, adjuvant therapy, the Eastern Cooperative Oncology Group Scale (ECOG) score, comorbidity, postoperative complications and stoma status.
Zhu^[13]^	Logistic regression mode	Single factor analysis, Multivariate analysis	234/859	Categorical variables	Age, sex, sleep disorders, postoperative radioiodine therapy, thyroid stimulating hormone (TSH) suppression level.
Dong^[14]^	Logistic regression mode	Single factor analysis, Multivariate analysis	78/218	Continuous variable	Times of radiotherapy/chemotherapy, PSQI, fertility needs, and tumor metastasis.

PSQI = Pittsburgh Sleep Quality Index.

**Table 3:** Performance and presentation of the model.

References	Performance of the model		Model validation	Model presentation
	Degree of discrimination	Degree of calibration		
Zou^[8]^	C-index = 0.828 (95% CI: 0.805–0.849)	The calibration curve	Bootstrap test	Nomogram model
Jia^[9]^	AUC = 0.850 (95% CI: 0.774–0.927), *P* < .001, Sensitivity = 0.912, Specific degrees = 0.698	The calibration curve	Bootstrap test	Nomogram model
Li^[10]^	C-index = 0.773 (95% CI: 0.692–0.854) AUC = 0.755, Sensitivity = 76.19%, Specific degrees = 66.67%	The calibration curve	The Hosmer–Lemeshow test	Nomogram model
Wang^[11]^	AUC = 0.685 (95% CI: 0.670–0.700)	_	Temporal validation	Formula of risk score obtained by partial regression coefficient of each factor
Hu^[12]^	AUC = 0.825 (95% CI: 0.764–0.887)	The calibration curve	Bootstrap test	Nomogram model
Zhu^[13]^	AUC = 0.720 (95% CI: 0.681–0.759)	The calibration curve	Bootstrap test	Nomogram model
Dong^[14]^	C-index = 0.885 (95% CI: 0.838–0.933)	The calibration curve	Bootstrap test	Nomogram model

### 3.3. Literature quality assessment

The PROBAST scale^[19]^ was used by 2 researchers to comprehensively assess the quality of the literature, and the evaluation results were carefully reviewed to ensure their accuracy. In the field of the research object, for research design is a retrospective study is a high risk of bias; in the field of predictors, all were at low risk of bias. In the field of data analysis, all were at high risk of bias (Table 4). The reasons for high risk of bias included: insufficient sample size in the Table 4 quality assessment of included literature modeling group; the occurrence of each independent variable frequency (events per variable) < 20 cases; the study will continuous variables into classification; the study was based on the single factor analysis of screening of variables; failing to report whether model calibration of test; internal validation was not reported in the literature. In the field, the results are of low risk of bias. In the field of predictors, all predictors are defined in the same way, but the outcome measures are not standardized at present, and a variety of tools for depression assessment have been adopted. All studies applicability good prediction model. The overall assessment showed that all studies had a high risk of bias, but the prediction model had good applicability.

**Table 4:** Quality assessment of included literature.

References	Risk of bias				Applicability			Overall	
	Participants	Predictors	Outcome	Analysis	Participants	Predictors	Outcome	Risk of bias	Applicability
Zou^[8]^	+	-	-	+	+	+	+	-	+
Jia^[9]^	+	-	-	+	+	+	+	-	+
Li^[10]^	+	-	-	+	+	+	+	-	+
Wang^[11]^	+	-	-	+	+	+	+	-	+
Hu^[12]^	+	-	-	+	+	+	+	-	+
Zhu^[13]^	+	-	-	+	+	+	+	-	+
Dong^[14]^	+	-	-	+	+	+	+	-	+

+ = low ROB/low concern regarding applicability.

- = high ROB/high concern regarding application.

### 3.4. Meta-analysis results

**Figure 2.**
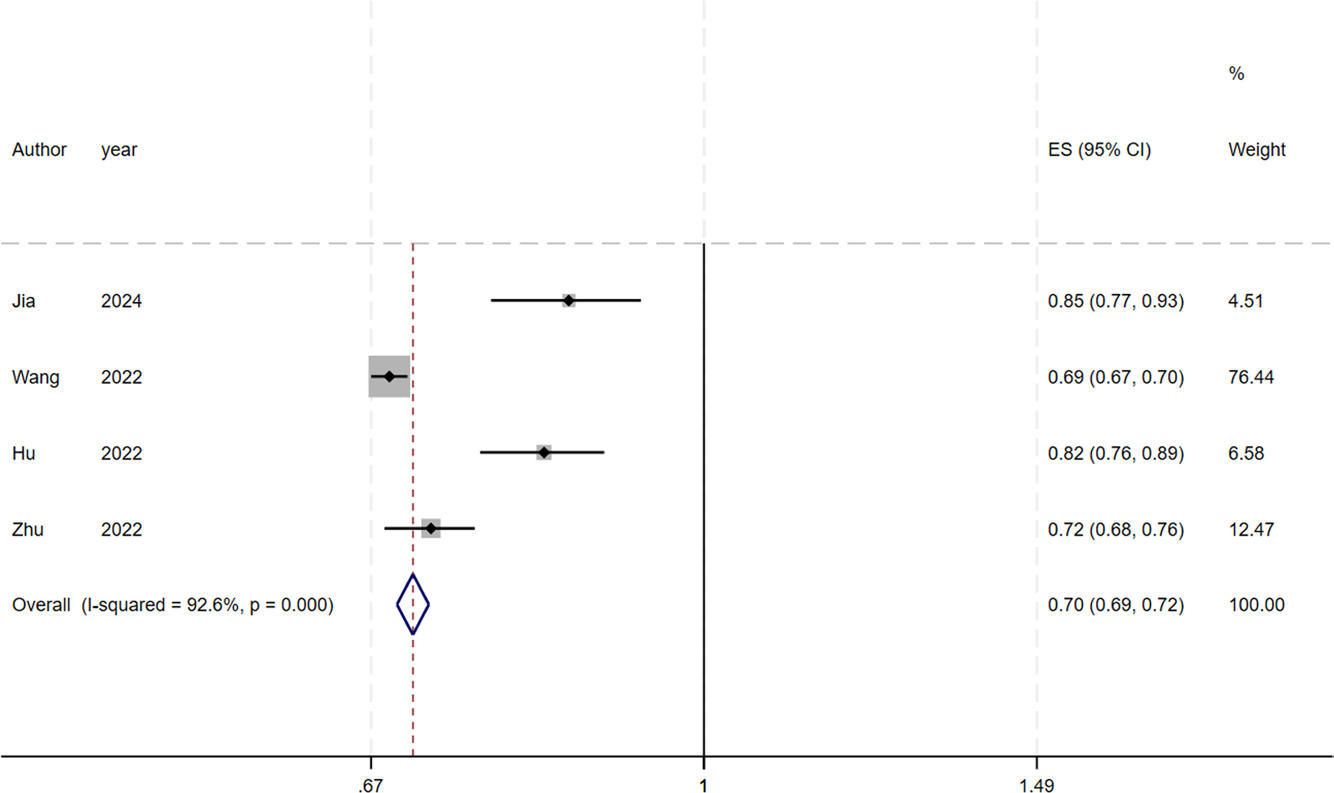
Forest plot of the random effects meta-analysis of pooled AUC estimates for 5 validation models. AUC = area under the curve.

**Figure 3.**
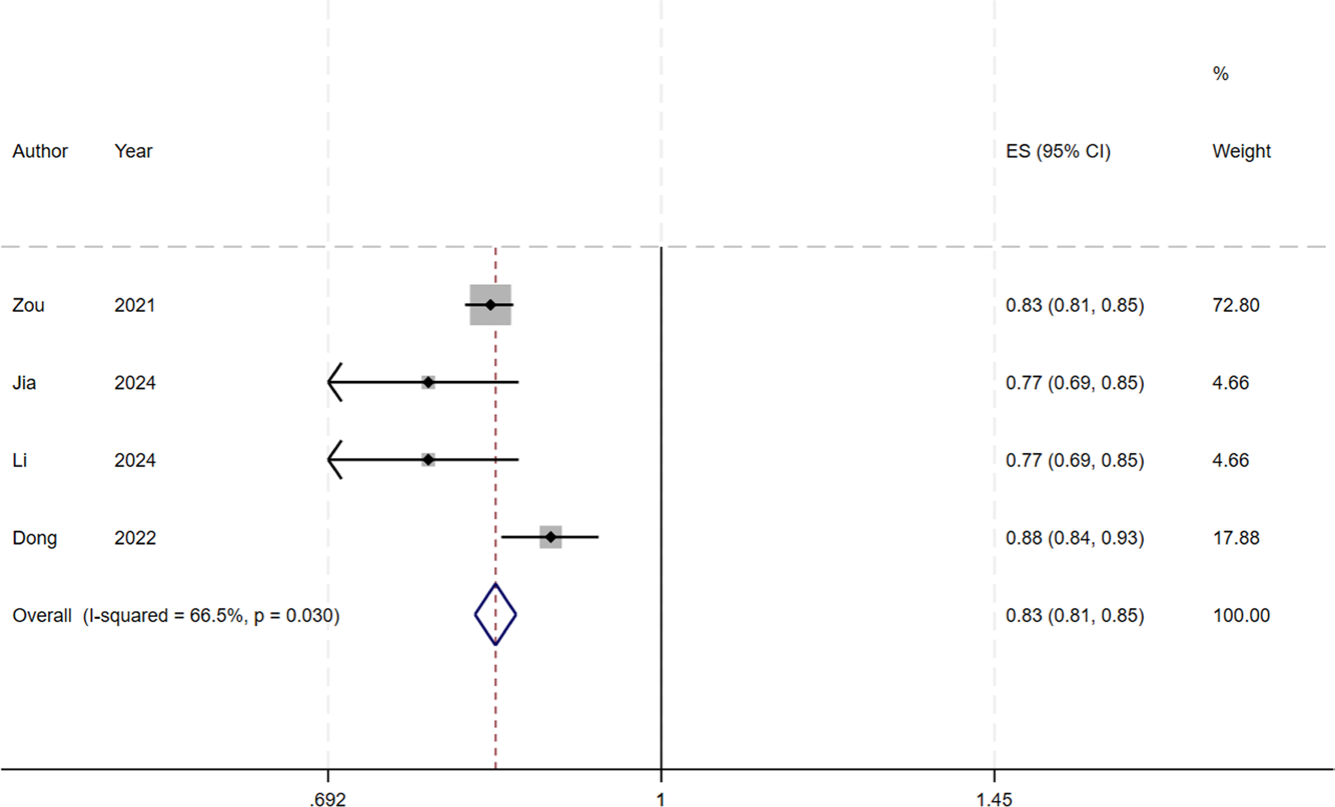
Forest plot of the random effects meta-analysis of pooled C-index estimates for 3 validation models.

**Figure 4.**
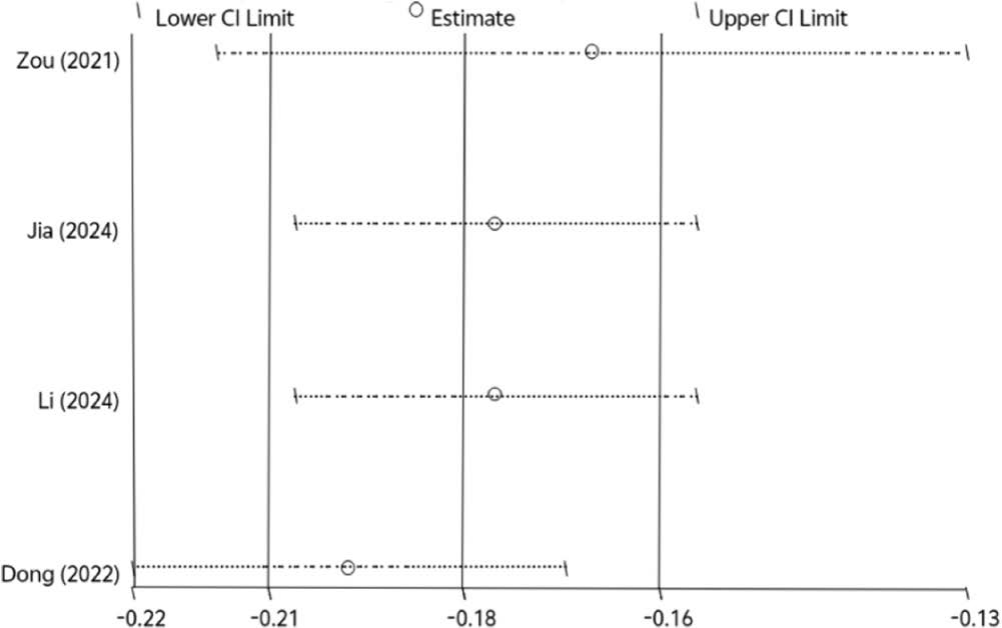
Sensitivity analysis of C-index.

**Figure 5.**
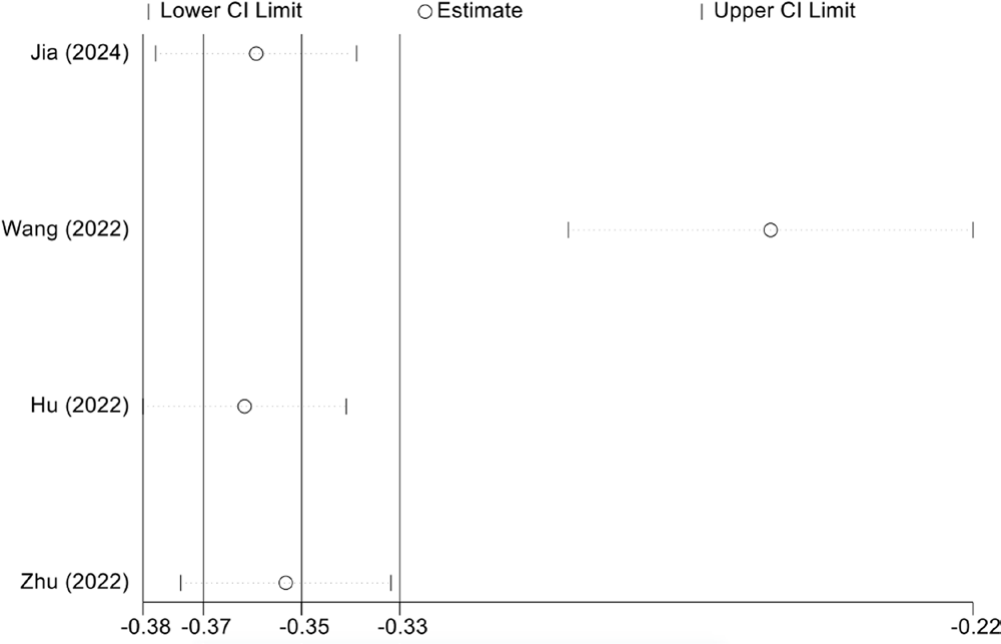
Sensitivity analysis of AUC. AUC = area under the curve.

Due to insufficient reporting of the details of the models in the included studies, 6 studies were eligible for meta-analysis of AUC and C-index. We include the use of the development of logistic regression model, the random effects model calculating AUC and C-index. Meta-analysis of AUC is 0.705 (95% CI: 0.691–0.718), I^2^ = 92.6% (*P* < .001), *P* < .0001, indicating that highly heterogeneity between studies (Fig. 2). The C-index was 0.833 (95% CI: 0.814–0.852), I^2^ = 66.5% (*P* = .030), *P* < .0001, indicating a high degree of heterogeneity among studies (Fig. 3). The Egger test result of AUC meta-analysis was *P* = .047 < .05, suggesting significant publication bias. The Egger test result of the C-index meta-analysis *P* = .725 > .05, suggesting no significant publication bias. Sensitivity analysis was performed on the results of C-index meta-analysis, as shown in Figure 4. After all literatures were eliminated, the combined results of the remaining studies were still statistically significant. Sensitivity analysis was performed on the results of meta-analysis of AUC, as shown in Figure 5. After all literatures were excluded, the combined results of the remaining studies were still statistically significant, and the heterogeneity of meta-analysis was mainly from the study of Wang^[11]^.

## 4. Discussion

Cancer survivors have a high incidence of depression, and cancer survivors’ depression-related psychological barriers can increase cancer-related pain, hinder the ability to respond effectively to the tumor, and even increase the risk of death.^[20]^ The depression occurrence prediction model has the ability for early detection of high-risk patients, can guide the staff to take preventive measures as soon as possible, and reduce the incidence of depression. A total of 7 risk prediction models for depression were included in this study. The modeling method was mainly logistic regression, and the model expression was mainly nomogram. Most of the models showed good prediction performance (AUC > 0.7), but there was a lack of research on model calibration evaluation and model validation.

Most of the depression occurrence models in this study had high discrimination, but the model construction, validation, and result reporting were not deeply optimized. Model build steps include: clear research direction, determining the source, variable selection, data preprocessing, etc. In terms of data sources, retrospective studies were used in all the prediction models included in this study. Due to the good representation of cohort studies, prospective data or registration data are recommended to be used as modeling data when optimizing the model in the future^[21]^ to reduce the risk of data bias.^[22]^ In terms of variable screening, most studies are based on univariate and multivariate logistic regression, which may increase the risk of wrong predictor selection.^[23]^ At present, studies put forward new variable selection methods,^[24]^ such as LASSO regression, Ridge regression, and ElasticNet regression, which can reduce the risk of excessive fitting; we suggest that future screening of variables can be combined with clinical practice to adopt new methods, to improve the accuracy of screening; Reporting and processing of missing data can avoid overfitting of the model,^[25]^ and future studies are recommended to improve the missing data handling.

When dealing with continuous variables, modeling continuous data into categorical variables may lead to excessive loss of model power. However, when the model is in the stage of clinical promotion, data transformation can be carried out to improve the convenience of researchers’ application.^[26]^ When the data type is continuous, the mean difference (MD) or standardized mean difference (SMD) is usually used as the effect statistic. Friedrich et al^[27]^ proposed another effect measurement method, namely the rational means (ROM). Experimental results show that ROM, MD, and SMD have similar statistical performance characteristics, and ROM can be used as a reasonable alternative to the continuous data mean difference method. For variables with continuous data, ROM can be used instead when the OR value is not obtained in the prediction model. Compared with the SMD effect size, ROM can better explain the impact of predictor variables on outcomes. In terms of performance evaluation, the core indicators included discrimination and calibration. Discrimination was often expressed by the AUC value or C-index. Calibration was evaluated by the H–L goodness-of-fit test or calibration curve. The lack of performance evaluation in the current study and the overfitting of the model inhibit the applicability of the model in a sense. The average sample size of the included studies was 388, which belonged to small sample studies. Lack of internal validation may lead to overestimation of the performance of the model, so internal validation is crucial. In addition, external validation can improve model promotion,^[28]^ but it needs modeling and test modules, according to a set of baseline comparisons. At present, some progress has been made in the research of prediction models, but further research is still needed in the aspects of model construction, validation, and reporting.

The existing prediction models reported in this review also have clinical implications. Tumor status was one of the predictors, and we found that tumor status included tumor stage and tumor metastasis. Tumor staging might tend to involve complex antitumor treatment, which may include not only major surgery, but also radiation and chemotherapy, etc. For example, stage I throat cancer patients often receive carbon dioxide laser therapy, while stage II, III, and IV patients are more likely to undergo partial/total laryngectomy, possibly followed by postoperative chemoradiotherapy. Patients with oral cancer also undergo facial reconstruction.^[29]^ The extent of surgery has a direct impact on psychological problems such as depression in head and neck cancer patients.^[9]^ Living with advanced metastatic cancer and receiving ongoing treatment can lead to psychological discomfort, as such patients experience adverse medical, psychological, and social consequences of cancer treatment, in addition to depression, fear, anxiety, uncontrollable uncertainty, and feelings of death. As a result, this group experiences high levels of related psychological distress such as depression.^[30–32]^ Previous studies have shown that^[33]^ tumor metastasis is positively correlated with depression in breast cancer patients, which is consistent with the above findings. The treatment of cancer survivors is another predictor, such as in prostate cancer survivors receiving castration, especially surgical castration. Physically, this can cause a sharp decline in testosterone levels. The testicular is the main producing organs of male hormones such as testosterone, which play an important role in regulating emotions and psychological states. After surgical castration, male patients may experience a physiological response to decreased testosterone levels, which results in mood changes such as mood swings, anxiety, and depression. Such physiological changes may trigger physical and psychological discomfort, including mood swings, insomnia, and fatigue, leading to anxiety and depression. In addition, castration treatment may lead to sexual dysfunction, such as erectile dysfunction and decreased libido, which may adversely affect the patient’s self-esteem and confidence, triggering anxiety and depression. In addition, castration treatment may lead to changes that affect the body image of the patient, such as weight gain and breast hypertrophy, which may affect one’s self-image and sense of self-identity, leading to anxiety and depression.^[14]^ Postoperative adjuvant therapy for cancer survivors is also a risk indicator of depression. Due to the uncertainty of treatment results, the application of adjuvant therapy may increase the economic burden and psychological pressure, which increases the risk of postoperative anxiety and depression in patients.^[34]^ A survey in South Korea^[35]^ showed that radiotherapy can increase the risk of anxiety and depression in breast cancer survivors. The studies included in this review also showed that antitumor treatments such as chemotherapy/radiotherapy can have negative effects on the psychological state of relevant cancer survivors, such as depression.^[14]^ Postoperative radioiodine therapy in survivors of thyroid cancer can increase the psychological distress of patients. Radioiodine therapy causes neck edema, pain, and reduced salivary gland function. At the same time, isolation is required during treatment, which increases patients’ fear, helplessness, loneliness, and depression.^[36]^ The baseline situation of cancer survivors is also a risk indicator of depression, which can be used as an early hint for clinical intervention, such as marital status, gender, age, etc. Studies have shown that men who are widowed have a higher risk of prostate cancer than men who are married or partnered. This could mean that marriage or a relationship with social support may help to promote a healthy lifestyle and positive attention to health care, which in turn affects the risk and prognosis of prostate cancer survivors, such as the fact that single or widowed prostate cancer survivors have higher risk of developing depression than those with partners.^[10]^ The association between age and the prevalence of depression in cancer survivors has been extensively explored in recent years, but the results have been inconsistent. Zhang et al and Nikbakhsh et al concluded that older cancer survivors are more prone to depression and anxiety.^[37,38]^ On the contrary, Tavoli et al^[39]^ and Walker et al found that older cancer survivors had lower levels of depression and anxiety, and Clark et al suggested that age was not a factor affecting the degree of depression and anxiety. Therefore, the role of age in the occurrence of depression and anxiety in cancer survivors needs to be further determined by more scholars. Depression is also associated with individual relationship status (married, partnered, single/divorced/widowed). Compared with married cancer survivors with partners, single, divorced, or widowed cancer survivors may lack emotional support because their primary emotional confidant is their spouse, while unmarried patients lack emotional support and the ability to cathartically cope with stress, leading to increased susceptibility to anxiety and depression.^[10,40]^ In cancer survivors, the study found that women were more prone to depression and other psychological distress. Men pay more attention to physical symptoms, while women are more sensitive and emotionally vulnerable, and their tolerance for cancer-stress events is lower than that of men, making them more prone to painful emotions. Surgery, postoperative complications, and adjuvant antineoplastic therapy often prevent motherhood, leading to loss of control and feelings of guilt.^[10–13,41]^ In previous univariate analyses of factors associated with depression among colorectal cancer survivors, an income of less than $30,000 was positively associated with screening for depression (OR 2.20, 95% CI 1.57–3.08, *P* < .001). Low income was an independent risk factor for depression (OR 1.50, 95% CI 1.02–2.22, *P* = .04). A good economic situation is a guarantee to meet the medical needs of patients.^[42]^ The cost of antitumor treatment for cancer survivors is high, and patients with low income need to bear significant economic pressure during the treatment process, which increases the psychological burden of patients and leads to anxiety and depression.^[43]^ Fertility needs are an independent risk factor for the development of psychological disorders in gynecological cancer survivors, as has also been confirmed in past studies.^[44]^ The physiology of gynecological cancer survivors is in a special state, and fertility is regarded as a need for female self-realization, which subtly affects the psychological state of patients.^[8]^ As a physiological need, sleep is an important biological regulation mechanism of the human body, which is related to the energy reserve and tissue repair of various functional organs. Sleep disorders increase daytime fatigue, reduce attention, work status, social function and quality of life, disturb physiological rhythm, and seriously affect the physical and mental health of patients.^[45]^ In recent years, sleep quality has become the main focus of research on cancer survivors. Sleep quality is an independent risk factor for psychological disorders in cancer survivors.^[13]^ Aquil et al^[46]^ confirmed that the Pittsburgh Sleep Quality Index was positively correlated with anxiety and depression. Armer et al^[47]^ showed that sleep quality is a risk factor for anxiety and depression in the first year of ovarian cancer survival. This suggests that it is necessary for clinical medical workers to improve the sleep quality of patients and reduce their psychological disorders.

The postoperative status of cancer survivors was also a predictor of the development of depression. Jia study results showed that patients with tracheal tubes were more likely to develop depression.^[9]^ For patients undergoing partial/total laryngectomy for laryngeal cancer, they need to carry the tracheal cannula for a long time, or even lifelong, and exposure of the tracheal cannula can make patients feel ashamed, making them reluctant to socialize.^[48]^ Therefore, nursing staff should strengthen health education for patients with tracheal tubes, help them to view positively the appearance changes brought by the tracheal tube, and relieve negative emotions. The functional status score of head and neck cancer survivors is an independent risk factor for depression. Patients with a Functional Assessment of Cancer Therapy-Head & Neck score < 20 are prone to depression and other psychological disorders, which is similar to the results of Henry et al.^[49]^ Due to the special location of the head and neck, patients often face speech and eating problems after treatment. The study found that patients with speech and eating disorders are more likely to avoid social activities and show more severe symptoms of mental disorders such as depression than those with only simple appearance changes.^[50]^ The physical pain of cancer survivors is also a predictor of depression. The results of Li study showed that the postoperative pain (Visual Analogue Scale) score of prostate cancer survivors is a predictor of adverse moods such as depression. Physical pain seriously affects the quality of daily life of cancer survivors, and the stress response caused by pain can induce anxiety and depression.^[51]^ For cancer survivors, especially in thyroid cancer survivors, the TSH suppression level is one of the important influencing factors of depression and other psychological distress. Due to excessive exogenous thyroid hormone causing subclinical thyroid hyperfunction, it causes harm to the cardiovascular system and skeletal system, leading to arrhythmia, reduced bone mineral density, and increased risk of heart failure and fracture, as well as symptoms such as thinness, palpitations, and agitation.^[52]^ Colostomy is a common treatment for patients with colorectal cancer. Previous studies have shown that colorectal cancer survivors with colostomy have higher levels of depression and anxiety than those without colostomy, possibly because colostomy surgery is associated with loss of normal anal function, distorted body image, and changes in personal care.^[53]^

Complications from antitumor therapy are predictors of depression, and previous large sample studies (1785 cases) showed that having 2 or more comorbidities were associated with depression in colorectal cancer survivors.^[10–13,54,55]^ A study included 2552 breast cancer survivors and found that the presence of cardiovascular disease had a negative impact on the quality of life, thus leading to anxiety and depression.^[56,57]^

A considerable number of studies have been conducted to predict the occurrence of depression by clinical presentation or baseline characteristics of cancer survivors. Wang study^[11]^ extracted the methylation data from the GEO database, calculated the statistical difference between the promoter of each gene and the DNA methylation (SIMPO) score of other body regions based on the DNA methylation data, and then re-selected the genes with high correlation with MDD. By combining the selected genes, SIMPO proposed the methylation depression index, and the stepwise regression method and random forest were used to construct the prediction model for breast cancer with an AUC range of 0.70 to 0.67. While this review systematically synthesizes the current research on prediction models for depression risk in cancer survivors, several limitations warrant acknowledgments: Literature Search Scope: the inclusion of only Chinese and English publications may have omitted significant studies published in other languages, introducing potential publication bias and geographic representation bias. Heterogeneity and methodological quality of included studies: the included studies exhibited substantial heterogeneity in cancer types, definitions and assessment tools for depression, study design (all retrospective), sample sizes, predictor selection, model development, and validation methodologies. This heterogeneity complicated comparative analysis and synthesis, potentially limiting the generalizability of this review’s conclusions. Furthermore, inadequate reporting of methodological quality (e.g., data preprocessing, handling of missing values, variable selection strategies, sufficiency of validation) in some studies hinders the assessment of their results’ reliability. Insufficient model validation and clinical applicability assessment: as highlighted, existing models generally lack rigorous internal validation and, critically, independent external validation. This raises concerns about their generalizability to real-world settings, diverse populations, and varying healthcare contexts, thereby limiting their immediate clinical utility. Moreover, both the primary studies and this review provided limited assessment of models’ clinical utility (e.g., decision curve analysis) and practical deployment feasibility (e.g., usability, cost-effectiveness). Inadequate depth and dynamic consideration of predictors: current models predominantly rely on static baseline variables (e.g., demographics, tumor characteristics, treatment modalities). There remains insufficient integration of dynamic changes occurring during treatment (e.g., fluctuations in pain, progression of treatment side effects, evolving social support, accumulating financial burden), psychosocial factors (e.g., stigma, coping styles, post-traumatic stress symptoms), and biomarkers (e.g., methylation-based biomarkers as mentioned, though not yet mature). This limitation likely constrains predictive accuracy and timeliness. Lack of model comparison and integration studies: this review identified multiple models, but found a scarcity of studies directly comparing these models within the same cohort or using standardized benchmarks. Furthermore, research on integrating the strengths of different models or developing more adaptable dynamic prediction models is currently lacking.

Despite these limitations, this review holds significant theoretical and practical value and identifies key directions for future research: emphasis on early identification: it provides clear evidence supporting the clinical value of developing effective predictive tools for the early identification of cancer survivors at high risk of depression, thereby providing a rationale for implementing preventive psychological interventions. Synthesis of key predictors: it systematically summarizes a range of predictors consistently associated with depression in cancer survivors (e.g., advanced tumor stage, specific treatment modalities and their side effects, sociodemographic factors, pain, functional status, financial hardship, sleep disturbances). This offers concrete screening cues for clinicians to identify high-risk individuals during routine care. Revealing model potential and limitations: it highlights that while current models demonstrate some discriminative ability, their limitations in validation and applicability necessitate caution in clinical implementation. Prioritizing rigorously validated models or combining them with clinical judgment is recommended. Implications for health policy and resource allocation: identifying individuals at high risk for depression facilitates the optimized allocation of limited psychosocial support resources. Shifting the focus towards earlier intervention enables more precise management, potentially reducing the overall psychological burden, improving quality of life among cancer survivors, and indirectly impacting cancer prognosis and healthcare costs.

In conclusion, this systematic review of 7 risk prediction models for depression in cancer survivors found that the included models had a high risk of bias, but their applicability was acceptable. These models were still in the development stage, and the overall quality of the research needed to be further improved.

## Author contributions

**Conceptualization:** Haonan Xu, Xiaobo Du.

**Data curation:** Haonan Xu, Wenqiang Tang, Yuwen Liang, Kunyuan Gan, Xinlan Liu, Xiongxin Yang.

**Formal analysis:** Kunyuan Gan, Xinlan Liu, Xiongxin Yang.

**Funding acquisition:** Xiaobo Du.

**Investigation:** Haonan Xu, Wenqiang Tang.

**Methodology:** Haonan Xu, Wenqiang Tang, Yuwen Liang, Kunyuan Gan.

**Project administration:** Haonan Xu, Wenqiang Tang, Xiaobo Du.

**Supervision:** Xiaobo Du.

**Visualization:** Haonan Xu, Yuwen Liang, Kunyuan Gan, Xinlan Liu, Xiongxin Yang.

**Writing – original draft:** Haonan Xu, Wenqiang Tang, Yuwen Liang, Kunyuan Gan, Xinlan Liu, Xiongxin Yang, Xiaobo Du.

**Writing – review & editing:** Haonan Xu, Wenqiang Tang, Xiaobo Du.
